# Supplementary material for: Exhaled Hydrogen as a Marker of Intestinal Fermentation Is Associated with Diarrhea in Kidney Transplant Recipients
Source: J Clin Med. 2021 Jun 28;10(13):2854. doi: 10.3390/jcm10132854 (PMC8267713; doi:10.3390/jcm10132854)
Supplement: Supplementary file 1 [file jcm-10-02854-s001.zip › jcm-1247433-supplementary.pdf]

Table S1. Nutritional data

Nutrients	Total (n=199)	Exhaled H2, per group 1.0-6.9 ppm (n=68)	7.0-19.9 ppm (n=61)	≥20.0 ppm (n=70)
Energy, kcal	2053 (2465–3069)	2605 (2067–3449)	2445 (1987–2827)	2455 (2027–3050)
Protein, % of energy	16.2 ± 37.6	15.8 ± 3.2	16.3 ± 3.4	16.4 ± 2.9
Animal protein, g	64.5 ± 27.7	65.5 ± 27.8	63.0 ± 28.2	64.8 ± 27.5
Plant based protein, g	39.7 ± 18.8	40.9 ±19.6	36.2 ± 12.8	41.6 ±21.9
Lipids, % of energy	37.6 ± 5.9	38.6 ± 5.7	37.8 ± 5.6	36.5 ± 6.4
Carbohydrate, % of energy	42.0 ± 6.6	41.7 ± 6.7	41.6 ± 6.5	42.8 ± 6.6
Mono and disaccharides, g	121.3 ± 43.7	126.2 ± 45.4	124.9 ± 35.5	113.6 ± 47.7 [c]
Polysaccharides, g	149.9 ± 35.7	143.2 ± 38.7	144.3 ± 28.3	161.0 ± 36.3 [ab]
Fiber, g	26.5 ± 12.0	27.0 ± 12.3	28.8 ± 11.3	27.5 ± 12.0
Calcium, mg	1234 ± 604	1337 ± 661	1138 ± 525	1218 ± 605
Iron, mg	13.3 ± 5.0	13.5 ± 5.3	12.8 ± 4.3	13.5 ± 5.4
Zinc, mg	14.2 ± 5.5	14.5 ± 5.6	13.4 ± 4.8	14.6 ± 5.8
Vitamin D, mcg	5.2 ± 2.8	5.2 ± 2.6	4.9 ± 2.2	5.6 ± 3.3
Vitamin C, mg	97.3 ± 54.6	92.0 ± 62.7	104.8 ± 51.1	95.8 ± 48.8
Vitamin E, mg	17.9 ± 8.0	18.2 ± 8.3	16.9 ± 6.2	18.6 ± 9.1

n = 199 [a] vs G1 [b] vs G2 [c] trend vs 1 p=0.07. Data are presented as mean ± standard deviation or median with interquartile ranges (IQRs).

Table S2. Multivariate binary logistic regression results with exhaled H_2 (≥ or < 20 ppm) as dependent variable

Potential determinants	Regression coefficient	Wald X^2 value	*p* value	OR	95% CI of OR Lower	Upper
Polysaccharides intake, g	0.014	6.00	0.014	1.015	1.003	1.026
Proton pump inhibitor use	-0.389	0.90	0.344	0.678	0.303	1.517
Mono and disaccharides intake, g	-0.009	2.71	0.099	0.991	0.981	1.002
Tacrolimus trough levels, ug/L	0.088	0.77	0.381	1.092	0.897	1.328
Vitamin C intake, mg	-0.003	0.51	0.477	0.997	0.989	1.005
Total cholesterol, mmol/L	0.012	0.00	0.984	1.012	0.329	3.111
LDL-cholesterol, mmol/L	-0.096	0.22	0.882	0.909	0.256	3.223

n = 196. Abbreviations: OR, odds ratio; CI, confidence interval; LDL, low-density lipoprotein.
